# The triglyceride-glucose index modulates the association between diabetes duration and insulin resistance in type 2 diabetes: a large cross-sectional study

**DOI:** 10.3389/fendo.2026.1762526

**Published:** 2026-02-18

**Authors:** Ya Dong, Xiaohong Huang, Mingyu Hao, Haiyan Li, Dewen Yan

**Affiliations:** 1 Department of Endocrinology, Shenzhen Second People’s Hospital, the First Affiliated Hospital of Shenzhen University, Health Science Center of Shenzhen University, Shenzhen Clinical Research Center for Metabolic Diseases, Shenzhen Center for Diabetes Control and Prevention, Shenzhen, China; 2 Department of Endocrinology, Shenzhen Bao’an Shiyan People’s Hospital, Bao’an Clinical Institute of Shantou University Medical College, Shenzhen, China

**Keywords:** diabetes duration, homeostatic model assessment of insulin resistance (HOMA-IR), insulin resistance (IR), triglyceride-glucose index (TyG index), type 2 diabetes mellitus (T2DM)

## Abstract

**Background:**

The longitudinal impact of diabetes duration on the progression of insulin resistance (IR) in type 2 diabetes (T2DM) remains to be fully elucidated. It is also unclear whether this relationship is uniform across all patients or modified by specific metabolic factors.

**Objective:**

To investigate the association between diabetes duration and IR, and to identify critical effect modifiers that define high-risk trajectories.

**Methods:**

This cross-sectional study enrolled 3,309 patients with T2DM, categorized into non-IR (HOMA-IR < 2.31, n = 1,530) and IR (HOMA-IR ≥ 2.31, n = 1,779) groups. Multivariable linear regression was used to assess the independent association between diabetes duration and HOMA-IR. Subgroup and interaction analyses were performed to identify effect modifiers. A two-piecewise linear regression model was employed to delineate threshold effects.

**Results:**

In the overall cohort, diabetes duration was independently associated with higher HOMA-IR after full adjustment (β = 0.04, 95% CI: 0.03-0.05, *P* < 0.001). This association was significant only in the IR group (β = 0.04, 95% CI: 0.02-0.06, *P* < 0.001) and absent in the non-IR group. Subgroup analysis revealed that the TyG index was the only significant effect modifier (β_interaction_ = 0.06, 95% CI: 0.02-0.09, *P*_interaction_ = 0.001). Crucially, a threshold effect was identified in the high-TyG group: HOMA-IR increased sharply by 0.08 per additional year of diabetes only after 4.0 years (*P* < 0.001).

**Conclusions:**

This study defines a novel, actionable high-risk phenotype in T2DM, characterized by a combination of a TyG index ≥ 9.187 and diabetes duration exceeding 4.0 years, which is associated with a markedly accelerated increase in IR. Given that the TyG index is adjustable, this framework provides a clinically meaningful, personalized strategy for targeted early intervention, enabling precise prevention for those most susceptible to worsening insulin resistance.

## 1 Introduction

Type 2 diabetes mellitus (T2DM) represents a rapidly escalating global public health burden. According to the International Diabetes Federation (IDF) Diabetes Atlas (11th edition, 2025) estimates for 2024, 589 million adults aged 20–79 years were living with diabetes worldwide, with projections rising to 853 million by 2050. In 2024, diabetes was responsible for approximately 3.4 million deaths among adults aged 20–79 years and an estimated USD 1.015 trillion in global health expenditure. An estimated 43% of adults living with diabetes are undiagnosed (1). As the predominant form of diabetes globally, T2DM accounts for the bulk of diabetes-related morbidity and disability (2), underscoring the importance of improved pathophysiological risk stratification and timely intervention.

At the individual level, T2DM is characterized by progressive beta-cell dysfunction and insulin resistance (IR) (3). IR, defined as an impaired biological response to insulin (4), is a fundamental pathophysiological defect that not only drives hyperglycemia but also contributes to a constellation of cardiometabolic complications, including hypertension, dyslipidemia, and cardiovascular disease (5, 6). The Homeostatic Model Assessment of Insulin Resistance (HOMA-IR) is a widely used method for estimating IR in clinical research (7). It is well-established that IR tends to worsen over time in many patients with T2DM (8–10), a phenomenon largely attributed to progressive beta-cell exhaustion (11) and the glucolipotoxic effects (12) of chronic hyperglycemia and elevated free fatty acids (13). However, the clinical trajectory of IR is highly heterogeneous: not all patients exhibit a linear or severe progression of IR with increasing diabetes duration (14). This variability suggests the presence of important modifying factors that may either exacerbate or mitigate the duration-related progression of IR.

The triglyceride-glucose (TyG) index, calculated from fasting triglyceride and glucose levels (15), has emerged as a robust, inexpensive, and easily accessible surrogate marker for IR (16). It correlates well with the gold-standard hyper insulinemic-euglycemic clamp and, in some populations, has shown superior predictive value for incident T2DM and cardiovascular events compared with HOMA-IR (17, 18). Mechanistically, the TyG index encapsulates the core concept of “glucolipotoxicity,” in which the synergistic adverse effects of hyperglycemia and dyslipidemia converge to impair insulin signaling (19, 20). Furthermore, recent studies have highlighted the role of the TyG index in stratifying patients with T2DM based on their risk of developing long-term complications such as cardiovascular disease and progressive IR (21).

We hypothesized that the TyG index, as an integrated marker of glucolipotoxicity, may serve not only as a reflector of IR severity but also as a critical modifier of the relationship between diabetes duration and IR progression. While the progression of IR over time is recognized, reliable and clinically actionable tools to predict which patients will experience rapid deterioration and when remain lacking. Identifying such a modifier is crucial for the early stratification of patients into distinct risk trajectories, thereby enabling personalized monitoring and intervention. Therefore, this large-scale cross-sectional study aimed to: 1) confirm the independent association between diabetes duration and HOMA-IR in a large T2DM cohort, and 2) investigate the potential modifying role of the TyG index in this critical relationship. Ultimately, this work seeks to evaluate the TyG index as a pragmatic tool for predicting nonlinear, high-risk IR trajectories, addressing a significant gap in clinical risk stratification.

## 2 Materials and methods

### 2.1 Study population

This cross-sectional study was conducted at the Department of Endocrinology, Shenzhen Second People’s Hospital, and enrolled 3,309 patients with type 2 diabetes (T2DM) between January 2017 and May 2022. This study was conducted in accordance with the Declaration of Helsinki and was approved by the Clinical Research Ethics Committee of Shenzhen Second People’s Hospital (Approval No.: 20220209005). The need for informed consent was waived by the same ethics committee due to the retrospective nature of the study based on an established database. The requirement for obtaining informed consent was waived by the committee due to the retrospective nature of the study and the use of anonymized clinical data. All patient information was de-identified to ensure confidentiality and privacy.

### 2.2 Inclusion criteria

The diagnosis of T2DM was based on the 2022 American Diabetes Association (ADA) criteria (22): fasting plasma glucose (FPG) ≥ 7.0 mmol/L, and/or 2-hour plasma glucose during an oral glucose tolerance test ≥ 11.1 mmol/L, and/or classic symptoms of hyperglycemia with a random plasma glucose ≥ 11.1 mmol/L. The etiologic type of diabetes was confirmed as T2DM according to the 1999 World Health Organization (WHO) classification system (23). Age ≥ 18 years in both genders.

### 2.3 Exclusion criteria

We excluded patients with the following conditions to minimize confounding: Type 1 diabetes, gestational diabetes, or other specific types of diabetes. Acute diabetic complications, including diabetic ketoacidosis or hyperosmolar hyperglycemic state. Severe hepatic dysfunction (alanine aminotransferase ≥ 5 times the upper limit of normal), alcoholic fatty liver disease, or autoimmune hepatitis. Stage 5 chronic kidney disease, defined as an estimated glomerular filtration rate (eGFR) < 15 mL/min/1.73m^2^ or on dialysis. Pancreatic diseases (e.g., glucagonoma, pancreatic tumor, acute pancreatitis). Clinical thyroid dysfunction (hyperthyroidism or overt hypothyroidism). Parathyroid disorders (hyperparathyroidism or hypoparathyroidism). Adrenal disorders (e.g., Cushing’s syndrome, Addison’s disease, pheochromocytoma, primary aldosteronism). Use of medications known to severely affect lipid metabolism (e.g., fibrates) or long-term glucocorticoid use. Incomplete data on FPG or fasting insulin (FINS), or outliers for HOMA-IR (values beyond mean ± 3 standard deviations).

### 2.4 Data collection and laboratory measurements

Clinical Data Collection: Demographic information, medical history, and medication use were collected using standardized questionnaires. Trained staff performed anthropometric measurements, including height, weight. Waist circumference (WC, cm) was measured using a non-stretchable tape at the midpoint between the lower margin of the last palpable rib and the top of the iliac crest at the end of a normal expiration. Hip circumference (HC, cm) was measured at the level of the greatest protrusion of the buttocks. The waist-to-hip ratio (WHR) was calculated as WC divided by HC. Hypertension was defined as systolic/diastolic blood pressure ≥ 140/90 mmHg or current use of antihypertensive medication. Diabetes duration was calculated from the initial date of diagnosis.

Laboratory Analyses: Following an overnight fast, venous blood samples were collected. Fasting plasma glucose (FPG), glycated hemoglobin (HbA1c), fasting insulin (FINS), and fasting lipid profiles [triglycerides (TG), total cholesterol (TC), low-density lipoprotein cholesterol (LDL-C), high-density lipoprotein cholesterol (HDL-C)], and white blood cell (WBC), neutrophil count, lymphocyte count, C-reactive protein (CRP), alanine aminotransferase (ALT), γ-glutamyl transferase (GGT), serum creatinine (Scr) were measured using standardized automated laboratory techniques.

Instrumental Examinations: All participants underwent a standardized ultrasonographic protocol. metabolic dysfunction-associated fatty liver disease (MAFLD) was assessed via liver ultrasound scanning.

Medication Assessment: The use of insulin was recorded.

### 2.5 Calculation of indices

HOMA-IR: was calculated as [FINS (μU/mL) × FPG (mmol/L)]/22.5. Patients were stratified into a non-IR group (HOMA-IR < 2.31) and an IR group (HOMA-IR ≥ 2.31) based on the cut-off point proposed by Xiang et al. (24) in a study of the Chinese population.

TyG Index: The index was calculated as the natural logarithm (Ln) of [TG (mg/dL) × FPG (mg/dL)/2].

Body mass index (BMI): BMI was calculated as weight in kilograms divided by the square of height in meters (kg/m^2^).

### 2.6 Statistical analyses

All statistical analyses were performed using R software (version 4.5.0). Categorical variables are presented as counts and percentages, and between-group comparisons were conducted using the chi-square test. Continuous variables were first assessed for normality. Normally distributed variables are expressed as mean ± standard deviation (SD) and compared using the independent-samples t test; non-normally distributed variables are presented as median (interquartile range, IQR) and compared using the Mann-Whitney U test.

Multivariable Regression Model Construction: To identify factors associated with IR among patients with T2DM, univariable linear regression analyses were first performed in the overall study population. Multivariable linear regression models were then built to focus on the association between diabetes duration and HOMA-IR. Three models were constructed sequentially: Model 1 (unadjusted), Model 2 (partially adjusted), and Model 3 (fully adjusted). Covariates were selected using the change-in-estimate approach, in conjunction with statistical and clinical considerations. Specifically, variables were included if they met the following criteria: ① statistically significant in univariable linear regression; ② inclusion or removal of the variable resulted in a ≥ 10% change in the regression coefficient (β) for diabetes duration; ③ low multicollinearity (variance inflation factor [VIF] < 10), or the variable had established or plausible clinical relevance. Overall model fit was evaluated using the adjusted coefficient of determination (adjusted R^2^). All regression coefficients (β) in both univariable and multivariable linear regression models were estimated using ordinary least squares (OLS), and the corresponding 95% confidence intervals were reported.

Sensitivity and Subgroup Analyses: To further assess the robustness of the association between diabetes duration and HOMA-IR across different insulin resistance states, multivariable linear regression analyses were performed separately in the IR group and the non-IR group. Additional subgroup analyses were conducted within the IR group. In subgroup analyses, age (60 years), BMI (< 24 kg/m^2^), and HbA1c (< 7%) were stratified using clinically meaningful cutoffs. Other continuous variables, including CRP, GGT, and the TyG index, were stratified by their median values. Categorical subgroup variables included gender, DN, MAFLD, and insulin use. All stratified and subgroup analyses were based on the fully adjusted model (Model 3).

Threshold Effect Analysis: To explore potential non-linear relationships between diabetes duration and HOMA-IR within TyG strata, participants were categorized into a high TyG group (≥ 9.187) and a low TyG group (< 9.187) according to the median TyG value (9.187). Within each TyG group, a two-piecewise linear regression model was fitted to examine the association between diabetes duration and HOMA-IR. The inflection point (ψ) was automatically estimated using the segmented package in R. In a two-segment piecewise linear regression model, the breakpoint location (ψ) and the segment-specific regression coefficients (β) before and after the breakpoint are jointly estimated using an iterative algorithm within the OLS framework. To evaluate the robustness of the inflection point estimate, bootstrap resampling (500 iterations) was used to obtain the 95% CI for breakpoint location (ψ). A likelihood ratio test (LRT) was performed to compare the two-piecewise model with the single-slope linear regression model and to determine whether the segmented model significantly improved model fit.

All statistical tests were two-sided, and a *P* value < 0.05 was considered statistically significant.

## 3 Results

### 3.1 Baseline characteristics of the study population

The baseline characteristics of the 3,309 participants, stratified by a HOMA-IR cut-off of 2.31, are presented in **Table 1**. The cohort comprised 1,530 (46.2%) individuals in the IR group (HOMA-IR ≥ 2.31) and 1,779 (53.8%) in the non-IR group. The median HOMA-IR was 4.37 (3.15-6.45) for the IR group, and 1.30 (0.87-1.71) for the non-IR group. Compared to the non-IR group, participants in the IR group exhibited a more pronounced adverse metabolic profile, characterized by significantly higher measures of adiposity (BMI, WC, HC, WHR), poorer glycemic control (HbA1c, FPG, FINS), and a more atherogenic lipid profile (TG, TC, LDL-C, HDL-C). Additionally, the IR group had a higher proportion of women, a longer median diabetes duration, a greater use of insulin, and elevated markers of systemic inflammation (WBC, neutrophil count, lymphocyte count, CRP) and liver enzymes (ALT, GGT). The prevalence of diabetic complications (DR, DPN, DN) and comorbidities (hypertension, coronary heart disease, MAFLD) was also significantly higher in the IR group (all *P* < 0.001), whereas no significant differences were observed in age or Scr. Consistent with these findings, the TyG index was significantly elevated in the IR group (9.23 ± 0.75 *vs*. 8.83 ± 0.71, *P* < 0.001).

**Table 1:** Baseline characteristics of patients in the IR group and non-IR group.

Characteristic	IR N = 1779	non-IR N = 1530	*P* value
Age (years)	58.00 (49.00, 67.00)	57.00 (50.00, 64.00)	0.138
Gender, n (%)			< 0.001
Male	1119 (62.90)	1059 (69.22)	
Female	660 (37.10)	471 (30.78)	
Diabetes duration (years)	10.00 (4.75, 15.00)	8.00 (4.00, 13.00)	< 0.001
BMI (kg/m^2^)	25.10 (23.10, 27.34)	24.16 (22.20, 26.25)	< 0.001
WC (cm)	92.00 (86.00, 99.00)	90.00 (84.00, 96.00)	< 0.001
HC (cm)	97.00 (93.00, 102.00)	96 (92.00, 100.00)	< 0.001
WHR	0.94 (0.90, 0.98)	0.93 (0.89, 0.97)	< 0.001
FPG (mmol/L)	7.30 (5.83, 9.30)	5.84 (5.00, 6.92)	< 0.001
FINS (IU/mL)	14.10 (10.30, 19.41)	4.55 (3.22, 6.40)	< 0.001
HOMA-IR	4.37 (3.15,6.45)	1.30 (0.87,1.71)	< 0.001
HbA1c (%)	9.30 (7.80, 11.00)	7.30 (6.50, 8.80)	< 0.001
WBC (10^9^/L)	6.64 (5.62, 7.84)	6.22 (5.30, 7.44)	< 0.001
Neutrophil count (10^9^/L)	3.84 (3.13, 4.76)	3.68 (2.91, 4.50)	< 0.001
Lymphocyte count (10^9^/L)	1.99 (1.58, 2.45)	1.88 (1.51, 2.28)	< 0.001
CRP (mg/L)	1.25 (0.50, 3.21)	0.65 (0.50, 1.93)	< 0.001
ALT (U/L)	18.00 (12.70, 27.40)	15.95 (11.70, 23.90)	< 0.001
GGT (U/L)	26.00 (19.00, 40.32)	22.00 (16.00, 33.00)	< 0.001
Scr (μmol/L)	64.70 (53.05, 79.00)	65.90 (55.80, 77.50)	0.186
TG (mmol/L)	1.67 (1.14, 2.48)	1.37 (0.93, 2.04)	< 0.001
TyG index	9.19 (8.70, 9.71)	8.79 (8.34, 9.23)	< 0.001
TC (mmol/L)	4.34 (3.56, 5.15)	4.17 (3.45, 4.86)	< 0.001
LDL-C (mmol/L)	2.70 (2.04, 3.36)	2.57 (1.95, 3.15)	< 0.001
HDL-C (mmol/L)	1.04 (0.90, 1.21)	1.09 (0.93, 1.27)	< 0.001
DR, n (%)			< 0.001
No	897 (50.42)	1024 (66.92)	
Yes	882 (49.58)	506 (33.08)	
DN, n (%)			< 0.001
No	1280 (71.95)	1225 (80.07)	
Yes	499 (28.05)	305 (19.93)	
DPN, n (%)			< 0.001
No	668 (37.55)	691 (45.16)	
Yes	1111 (62.45)	839 (54.84)	
Hypertension, n (%)			< 0.001
No	893 (50.20)	862(56.34)	
Yes	886 (49.80)	668 (43.66)	
Coronary heart disease, n (%)			0.011
No	1535 (86.28)	1365 (89.22)	
Yes	244 (13.72)	165 (10.78)	
MAFLD, n (%)			< 0.001
No	677 (38.06)	739 (48.30)	
Yes	1102 (61.94)	791 (51.70)	
Insulin use, n (%)			< 0.001
No	460 (25.86)	780 (50.98)	
Yes	1319 (74.14)	750 (49.02)	

IR, insulin resistance; BMI, body mass index; WC, waist circumference; HC, hip circumference; WHR, waist-to-hip ratio; FPG, fasting plasma glucose; FINS, fasting insulin; HOMA-IR, homeostasis model assessment for insulin resistance; HbA1c, hemoglobin A1c; WBC, white blood cell; CRP, C-reactive protein; ALT, alanine aminotransferase; GGT, γ-glutamyl transferase; Scr, serum creatinine; TG, triglyceride; TyG index, triglyceride-glucose index; TC, total cholesterol; LDL-C, low-density lipoprotein-cholesterol; HDL-C, high-density lipoprotein cholesterol; DR, diabetic retinopathy; DN, diabetic nephropathy; DPN, diabetic peripheral neuropathy; MAFLD, metabolic dysfunction-associated fatty liver disease.

### 3.2 Univariate analysis of factors associated with insulin resistance

As shown in **Table 2**, univariate analysis revealed that HOMA-IR demonstrated significant positive associations with age, diabetes duration, measures of adiposity (BMI, WC, HC, WHR), glycemic parameters (HbA1c, FPG, FINS), lipid profiles (TyG index, TG, TC, LDL-C), liver function (ALT, GGT), renal function (Scr), and inflammatory markers (WBC, neutrophil count, lymphocyte count, CRP). Conversely, HDL-C was inversely correlated with HOMA-IR. Furthermore, several clinical conditions, including female sex, MAFLD, hypertension, coronary heart disease, DR, DN, and DPN, were also significantly associated with elevated HOMA-IR values.

**Table 2:** Univariate analysis of factors associated with HOMA-IR.

Variables	β (95% CI)	*P* value
Age (years)	0.01 (0.01, 0.02)	0.001
Female	0.64 (0.44,0.85)	< 0.001
Diabetes duration (years)	0.06 (0.05, 0.08)	< 0.001
BMI (kg/m^2^)	0.13 (0.10, 0.15)	< 0.001
WC (cm)	0.05 (0.04, 0.06)	< 0.001
HC (cm)	0.04 (0.03, 0.05)	< 0.001
WHR	6.65 (5.12, 8.17)	< 0.001
HbA1c (%)	0.50 (0.46, 0.54)	< 0.001
WBC (10^9^/L)	0.19 (0.13, 0.24)	< 0.001
Neutrophil count (10^9^/L)	0.21 (0.13, 0.28)	< 0.001
Lymphocyte count (10^9^/L)	0.32 (0.17, 0.47)	< 0.001
CRP (mg/L)	0.02 (0.01, 0.03)	< 0.001
ALT (U/L)	0.01 (0.00, 0.01)	0.004
GGT (U/L)	0.00 (0.00, 0.01)	0.001
Scr (μmol/L)	0.01 (0.00, 0.01)	0.001
TG (mmol/L)	0.15 (0.10, 0.20)	< 0.001
TyG index	1.21 (1.08, 1.33)	< 0.001
TC (mmol/L)	0.16 (0.08, 0.25)	< 0.001
LDL-C (mmol/L)	0.21 (0.10, 0.31)	< 0.001
HDL-C (mmol/L)	-0.78 (-1.12, -0.43)	< 0.001
DR	1.08 (0.88, 1.27)	< 0.001
DN	0.80 (0.57, 1.02)	< 0.001
DPN	0.50 (0.30, 0.70)	< 0.001
Hypertension	0.45 (0.25, 0.65)	< 0.001
Coronary heart disease	0.54 (0.24, 0.83)	< 0.001
MAFLD	0.60 (0.40, 0.80)	< 0.001
Insulin use	1.64 (1.44,1.83)	< 0.001

HOMA-IR, homeostasis model assessment for insulin resistance; BMI, body mass index; WC, waist circumference; HC, hip circumference; WHR, waist-to-hip ratio; FPG, fasting plasma glucose; FINS, fasting insulin; HbA1c, hemoglobin A1c; WBC, white blood cell; CRP, C-reactive protein; ALT, alanine aminotransferase; GGT, γ-glutamyl transferase; Scr, serum creatinine; TG, triglyceride; TyG index, triglyceride-glucose index; TC, total cholesterol; LDL-C, low-density lipoprotein-cholesterol; HDL-C, high-density lipoprotein cholesterol; DR, diabetic retinopathy; DN, diabetic nephropathy; DPN, diabetic peripheral neuropathy; MAFLD, metabolic dysfunction-associated fatty liver disease.

### 3.3 Independent association between diabetes duration and HOMA-IR

In this section, we examined the association between diabetes duration and HOMA-IR across three models with varying levels of adjustments. The regression coefficients (β), 95% CI, and *P*-values for each model, as well as the adjusted R^2^ values, are summarized in **Table 3**.

**Table 3:** Relationship between diabetes duration and HOMA-IR in patients with T2DM across different models.

Variables	Model1			Model2			Model3		
	β (95% CI)	*P* value	Adj_R^2^	β (95% CI)	*P* value	Adj_R^2^	β (95% CI)	*P* value	Adj_R^2^
Diabetes duration (years)	0.06 (0.05,0.08)	<0.001	0.02	0.07 (0.06,0.09)	<0.001	0.07	0.04 (0.03,0.05)	<0.001	0.33

HOMA-IR, homeostasis model assessment for insulin resistance; T2DM, Type 2 Diabetes Mellitus; β, regression coefficient; CI, confidence interval; Adj_R2, adjusted R^2^.

Model 1 (Unadjusted Model): In Model 1, diabetes duration was positively associated with HOMA-IR (β = 0.06, 95% CI: 0.05-0.08, *P* < 0.001), explaining 2% of the variation in HOMA-IR (adjusted R^2^ = 0.02).

Model 2 (Partially Adjusted Model): After adjusting for age, gender, BMI, and WC, the association between diabetes duration and HOMA-IR remained significant (β = 0.07, 95% CI: 0.06-0.09, *P* < 0.001), with an increased adjusted R^2^ of 0.07, indicating a better fit compared to the unadjusted model (**Table 3**). Notably, the variables of gender and WC also exhibited significant associations with HOMA-IR (**Supplementary Table 1**).

Model 3 (Fully Adjusted Model): Further adjustment for additional clinical factors (including HbA1c, TyG index, lymphocyte count, DR, MAFLD, insulin use, HDL-C, ALT, GGT, CRP, neutrophil count, hypertension, coronary heart disease, DN, TG, WBC, DPN, and Scr) yielded a somewhat reduced, yet still statistically significant, association (β = 0.04, 95% CI: 0.03-0.05, *P* < 0.001). The adjusted R^2^ for this model was 0.33, indicating that it explains approximately 33% of the variance in HOMA-IR. The significant predictors of HOMA-IR include WC, HbA1c, the TyG index, insulin use, coronary heart disease, and TG, among others, which collectively improved the model fit and highlighted their contribution to the observed association (**Supplementary Table 1**).

### 3.4 Stratified analysis by insulin resistance status

After stratifying participants by baseline IR status, diabetes duration showed heterogeneous associations with HOMA-IR across strata (**Table 4**). In the non-IR group, diabetes duration was not significantly associated with HOMA-IR in the crude model (Model 1: β = -0.00, 95% CI: -0.01-0.00, *P* = 0.642, adjusted R^2^ = 0.00), in the partially adjusted model (Model 2: β = 0.00, 95% CI: -0.00-0.01, *P* = 0.072, adjusted R^2^ = 0.09), or in the fully adjusted model (Model 3: β = 0.00, 95% CI: -0.00-0.01, *P* = 0.211, adjusted R^2^ = 0.17). In contrast, in the IR group, longer diabetes duration was consistently associated with higher HOMA-IR in Model 1 (β = 0.05, 95% CI: 0.04-0.07, *P* < 0.001, adjusted R^2^ = 0.02), Model 2 (β = 0.05, 95% CI: 0.03-0.07, *P* < 0.001, adjusted R^2^ = 0.04), and Model 3 (β = 0.04, 95% CI: 0.02-0.06, *P* < 0.001, adjusted R^2^ = 0.18). These findings indicate that the positive association between diabetes duration and HOMA-IR is mainly evident among participants with baseline insulin resistance.

**Table 4:** Association between diabetes duration and HOMA-IR, stratified by baseline insulin resistance status.

Variables	Model1			Model2			Model3		
	β (95% CI)	*P* value	Adj_R^2^	β (95% CI)	*P* value	Adj_R^2^	β (95% CI)	*P* value	Adj_R^2^
non-IR group									
Diabetes duration (years)	-0.00 (-0.01,0.00)	0.642	0.00	0.00 (-0.00,0.01)	0.072	0.09	0.00 (-0.00,0.01)	0.211	0.17
IR group									
Diabetes duration (years)	0.05 (0.04,0.07)	<0.001	0.02	0.05 (0.03,0.07)	<0.001	0.04	0.04 (0.02,0.06)	<0.001	0.18

HOMA-IR, homeostasis model assessment for insulin resistance; β, regression coefficient; CI, confidence interval; Adj_R2, adjusted R^2^; IR, insulin resistance.

Model 2 was adjusted for age, sex, BMI, and WC. Model 3 was further adjusted for HbA1c, TyG index, lymphocyte count, DR, MAFLD, insulin use, and other prespecified clinical covariates (detailed coefficients for all covariates are provided in **Supplementary Table 2**).

### 3.5 The TyG index as an effect modifier

To examine potential effect modification, we performed prespecified subgroup analyses in the IR cohort (n = 1,779) using the fully adjusted Model 3 and formally tested interaction terms between diabetes duration and each stratification variable (**Table 5**). Age (< 60 *vs*. ≥ 60 years), BMI (< 24 *vs*. ≥ 24 kg/m^2^), and HbA1c (< 7% *vs*. ≥ 7%) were stratified using clinically meaningful cutoffs, whereas other continuous variables were dichotomized at their median values, including the TyG index (median cut-off: 9.187).

**Table 5:** Subgroup analysis of the association between diabetes duration and HOMA-IR in the IR cohort (N = 1779).

Variables	n	β_association_ (95% CI)	*P*_association_	Adj_R^2^	β_interaction_ (95% CI)	*P*_interaction_
Age (years)					-0.01 (-0.04,0.03)	0.602
< 60	982	0.03 (0.00, 0.06)	0.048	0.18		
≥ 60	797	0.04 (0.01, 0.06)	0.008	0.19		
Gender					0.01 (-0.02,0.05)	0.446
Male	1119	0.03(-0.00, 0.07)	0.063	0.19		
Female	660	0.03 (0.01, 0.06)	0.007	0.16		
BMI (kg/m^2^)					0.01 (-0.02,0.05)	0.421
< 24	626	0.04 (0.02, 0.07)	0.001	0.20		
≥ 24	1153	0.02 (-0.01, 0.05)	0.191	0.18		
HbA1c (%)					0.02 (-0.04,0.07)	0.555
< 7	180	0.03 (0.01, 0.05)	0.005	0.16		
≥ 7	1599	0.02 (-0.03, 0.08)	0.440	0.08		
CRP (mg/L)					0.01 (-0.02,0.04)	0.623
< 1.25	889	0.05 (0.01,0.08)	0.004	0.17		
≥ 1.25	890	0.03 (-0.00,0.05)	0.055	0.18		
GGT (U/L)					-0.00 (-0.04,0.03)	0.870
< 26	857	0.03 (0.00,0.06)	0.045	0.21		
≥ 26	922	0.04 (0.01,0.07)	0.006	0.16		
TyG index					0.06 (0.02,0.09)	0.001
< 9.187	889	0.06 (0.03, 0.10)	0.000	0.16		
≥ 9.187	890	0.02 (-0.01, 0.04)	0.184	0.06		
DN					-0.01 (-0.05,0.02)	0.518
Yes	499	0.04 (0.01,0.06)	0.002	0.18		
No	1280	0.04 (-0.01,0.08)	0.092	0.15		
MAFLD					0.01 (-0.03,0.04)	0.751
Yes	1102	0.03 (0.00, 0.06)	0.046	0.16		
No	677	0.04 (0.01, 0.07)	0.006	0.21		
Insulin use					0.04 (-0.01,0.08)	0.110
Yes	1319	0.01 (-0.03, 0.04)	0.780	0.15		
No	460	0.04 (0.02, 0.07)	0.001	0.16		

β_association_ and *P*_association_ indicates the effect estimate (regression coefficient) and significance of of the association between diabetes duration and HOMA-IR within each subgroup, βinteraction and Pinteraction indicate the effect estimate and significance of the interaction term between diabetes duration and the stratification variable.

HOMA-IR, homeostasis model assessment for insulin resistance; IR, insulin resistance; β, regression coefficient; CI, confidence interval; Adj_R2, adjusted R^2^; BMI, body mass index; HbA1c, hemoglobin A1c; CRP, C-reactive protein; GGT, γ-glutamyl transferase; TyG index, triglyceride-glucose index; DN, diabetic nephropathy; MAFLD, metabolic dysfunction-associated fatty liver disease.

Overall, no statistically significant interactions were observed for age, sex, BMI, HbA1c, CRP, GGT, DN, MAFLD, or insulin use (all *P*_interaction_ > 0.05). In contrast, the TyG index significantly modified the association between diabetes duration and HOMA-IR (*P_interaction_* = 0.001). Specifically, in the low TyG group (< 9.187; n = 889), diabetes duration was positively associated with HOMA-IR (β = 0.06, 95% CI: 0.03-0.10, *P* = 0.000). However, in the high TyG group (≥ 9.187; n = 890), no statistically significant positive correlation was observed (β = 0.02, 95% CI: -0.01-0.04; *P* = 0.184). This substantial difference reveals that the TyG level is a critical stratification factor influencing the core relationship between diabetes duration and HOMA-IR, warranting further in-depth investigation.

### 3.6 Threshold effect analysis: identifying a high-risk trajectory

**Figure 1:**
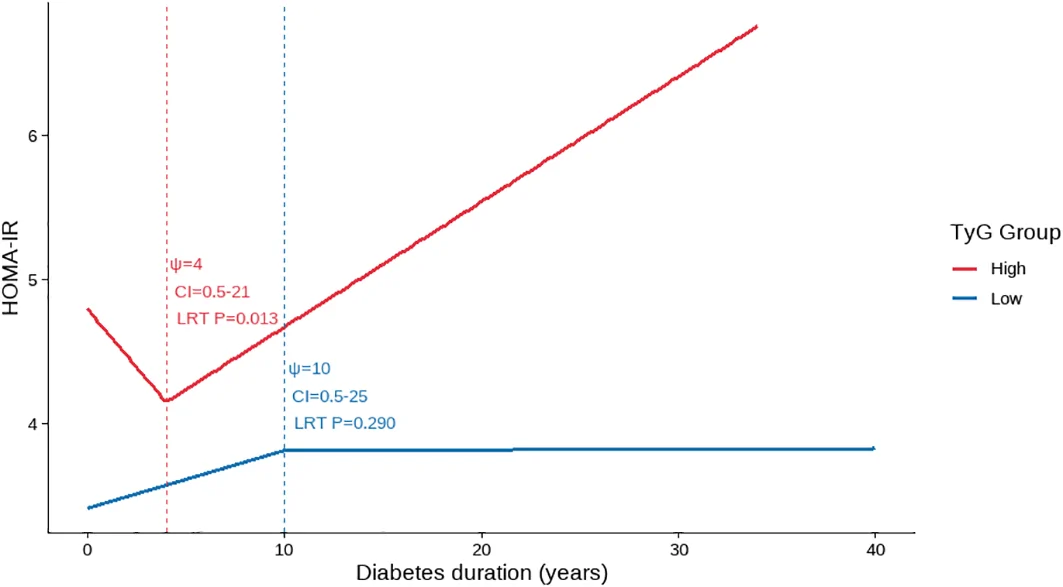
The relationship between diabetic duration and HOMA-IR in groups with different TyG index values. HOMA-IR, homeostasis model assessment for insulin resistance; TyG index, triglyceride-glucose index; ψ, inflection point; CI, confidence interval; LRT, likelihood ratio test.

To further clarify whether the association between diabetes duration and HOMA−IR was linear or exhibited a potential turning point, we conducted a threshold effect analysis using a two-piecewise linear regression model stratified by TyG index median (cut-off: 9.187) under the fully adjusted Model 3. LRT was used to compare the linear model with the two-piecewise linear regression model, and the estimated inflection point (ψ) is shown in **Table 6** and **Figure 1**.

**Table 6:** Threshold effect analysis of the relationship between the diabetic duration and HOMA-IR in different TyG index groups.

Variables	β (95%CI)	*P* value	Adj_R^2^
TyG index < 9.187			
Diabetes duration ≤ 10.0 years	-0.00 (-0.05,0.04)	0.832	0.08
Diabetes duration > 10.0 years	-0.01 (-0.05,0.04)	0.817	0.07
*P*_LRT_	0.290		
TyG index ≥ 9.187			
Diabetes duration ≤ 4.0 years	-0.08 (-0.33,0.16)	0.496	0.10
Diabetes duration > 4.0 years	0.08 (0.03,0.13)	<0.001	0.15
*P*_LRT_	0.013		

HOMA-IR, homeostasis model assessment for insulin resistance; TyG index, triglyceride-glucose index; β, regression coefficient; CI, confidence interval; Adj_R2, adjusted R^2^; LRT, likelihood ratio test.

In the low TyG group (TyG index < 9.187), the estimated turning point was approximately 10.0 years (ψ = 10, 95% CI: 0.5-25). However, the *P*_LRT_ did not support a significant improvement of the two-piecewise model over the linear model (*P*_LRT_ = 0.290). Consistently, the slopes on both sides of the candidate turning point were close to null and not statistically significant (≤ 10 years: β = -0.00, 95% CI: 0.05-0.04, *P* = 0.832; > 10 years: β = -0.01, 95% CI: -0.05-0.04, *P* = 0.817), suggesting no evidence of a meaningful threshold effect in this subgroup.

In contrast, in the high TyG group (TyG index ≥ 9.187), a statistically significant threshold effect was observed with an estimated turning point at approximately 4.0 years (ψ = 4, 95% CI: 0.5-21), and the two-piecewise model fit the data better than the linear model (*P*_LRT_ = 0.013). Before 4.0 years, diabetes duration was not significantly associated with HOMA-IR (β = -0.08, 95% CI: -0.33-0.16, *P* = 0.496). After 4.0 years, a longer diabetes duration was significantly associated with higher HOMA-IR (β = 0.08, 95% CI: 0.03-0.13, *P* < 0.001), indicating that individuals with a higher TyG index may enter a high-risk trajectory characterized by a progressive increase in IR beyond 4.0 years of diabetes duration (**Table 6** and **Figure 1**).

## 4 Discussion

In this large-scale cross-sectional study of patients with T2DM, we delineated a phenotype-dependent relationship between diabetes duration and insulin resistance (IR) with greater granularity. Our principal findings are threefold. First, we confirmed diabetes duration as an independent risk factor for worsening HOMA-IR in the overall cohort. Second, this association was not uniform: it was evident only among individuals with established IR, but absent in those without IR. Finally, and most notably, we identified the TyG index as the paramount effect modifier within the IR cohort and defined a specific high-risk trajectory characterized by a TyG index ≥ 9.187 and diabetes duration exceeding 4 years, beyond which IR escalates sharply.

### 4.1 The positioning within a precision-phenotyping framework

The independent positive association between diabetes duration and HOMA-IR in our overall cohort aligns with the natural history of T2DM, where progressive IR often occurs over time (25). However, our stratified analysis provides a crucial, refined insight: diabetes duration should not be treated as a universal surrogate of metabolic deterioration. Instead, the adverse metabolic meaning of “longer duration” depends on the patient’s underlying phenotype at assessment. In practical terms, our data suggest at least two broad phenotypes-those with preserved metabolic reserve (non-IR) and those with compromised reserve (IR)-and further partition the latter into distinct risk pathways using TyG. This means the non-IR individuals possess a greater metabolic reserve or compensatory capacity, potentially through preserved beta-cell function and adipocyte health, which buffers against the erosive effects of longer disease duration. Conversely, in the IR individuals the metabolic system may already be operating at a precarious threshold. Here, each additional year of diabetes may exacerbate underlying pathologies such as lipotoxicity, chronic inflammation, or mitochondrial dysfunction, leading to a measurable decline in insulin sensitivity (26–28). This critical heterogeneity shifts the clinical perspective from a one-size-fits-all model to one emphasizing early phenotyping, in which simple, routinely available markers can help identify individuals for intensified monitoring and targeted interventions.

### 4.2 The TyG index: a pivotal stratifier and potential driver of high-risk trajectories

The most salient finding of our study is the identification of the TyG index as the sole and highly significant effect modifier of the duration-IR relationship. Among numerous clinical and metabolic parameters, only the TyG index demonstrated a powerful interaction, effectively stratifying the IR cohort into two distinct risk pathways. The TyG index is a robust surrogate marker of IR (29) that integrates both lipid and glucose metabolism (30). A high TyG index signifies a state of pronounced lipid dysregulation (elevated triglycerides, often with low HDL-C) and glucose intolerance (31). This glucolipotoxic environment, characterized by chronic exposure of tissues to excessive circulating glucose and fatty acids or their derivatives, directly impairs insulin signaling through several interconnected mechanisms (12). These include mitochondrial dysfunction, where excess lipids overwhelm mitochondrial oxidative capacity, leading to oxidative stress and impaired insulin signaling (32). Additionally, the inability of adipose tissue to expand properly under glucolipotoxic conditions results in ectopic lipid deposition in non-adipose tissues, exacerbating insulin resistance (33). The accumulation of pro-inflammatory cytokines from senescent cells and macrophage infiltration in adipose tissue further activates inflammatory kinases like JNK and IKKβ, which disrupt insulin signaling (34). Endoplasmic reticulum (ER) stress, triggered by the accumulation of misfolded proteins in the ER, also impairs insulin response and promotes inflammation (35). In patients with a high TyG index, this toxic glucolipotoxic metabolic milieu, once it exceeded a critical duration, may “unmask” or amplify the cumulative adverse impact of prolonged diabetes exposure on IR (β = 0.07 in the high-TyG group *vs*. β = 0.04 in the overall IR group). In contrast, in the low-TyG group, the lower TyG profile may be associated with reduced glucolipotoxicity, thereby not exacerbating the duration-related worsening of insulin resistance.

The TyG cutoff value of 9.187 aligns with previous research that established pivotal TyG thresholds of 9.05 for all-cause mortality and 8.84 for cardiovascular disease mortality (36). Furthermore, supporting evidence indicates that a TyG index above 8.40 is a potent risk marker, where each standard deviation increase was associated with a 66% higher probability of major adverse cardiovascular events (37). Furthermore, another study showed elevated TyG index was significantly associated with coronary heart disease prevalence in hypertensive patients (38). Similarly, higher TyG levels were independently associated with increased stroke risk in a dose-dependent manner (39). Collectively, these findings suggest that the TyG index, particularly around the threshold of 9, serves as a robust integrator of metabolic risk, effectively linking the severity of insulin resistance to the consequent escalation in related diseases mortality risk.

### 4.3 The high-risk trajectory: clinical implications for precision medicine

Our threshold effect analysis significantly refines our understanding of diabetes progression, offering a clinically actionable insight through the lens of “precision phenotyping” in T2DM. The identification of a sharp inflection point at 4.0 years exclusively in the high-TyG group, but not in the low-TyG group, defines a clear high-risk trajectory. This phenotype-specific nonlinear progression indicates that for patients stratified into this high-TyG phenotype (TyG index ≥ 9.187), the first 4.0 years of diabetes may represent a critical metabolically stable yet high-risk window. However, beyond this threshold, the metabolic system reaches a tipping point, and IR increases precipitously at a rate of 0.08 HOMA-IR units per year-twice the average rate in the overall IR cohort. This nonlinear progression underscores the limitations of a one-size-fits-all approach to diabetes management and highlights the necessity of dynamic, phenotype-specific risk stratification.

Current clinical management of type 2 diabetes is primarily guided by individualized glycemic targets, body weight, and established cardiovascular, kidney, and other metabolic comorbidities, while simple and practical tools for prospectively identifying patients at particularly high risk of accelerated progression of insulin resistance are not routinely incorporated into current treatment algorithms (40, 41). This represents an important clinical gap, as accumulating evidence emphasizes that the early institution of targeted interventions is crucial for preserving beta-cell function and altering the long-term disease course (42). Our study provides a practical solution: the combined assessment of the TyG index and diabetes duration readily identifies this high-risk phenotype in a clinical setting. Specifically, when a patient demonstrates persistently elevated TyG values over follow-up and is approaching the 4.0-year window identified in our analyses, clinicians consider this profile as a high-risk phenotype for impending, rapid decline in insulin sensitivity. By using the TyG index, an accessible and practical biomarker, clinicians can achieve risk stratification in routine practice.

For the high-risk phenotype characterized by long-duration T2DM (≥ 4.0 years) combined with an elevated TyG index (TyG index ≥ 9.187), identified in this study, clinical management strategy must undergo a fundamental shift-from a traditional glucocentric approach to a proactive, pathophysiology-driven intervention aimed at disrupting the vicious cycle of glucolipotoxicity. Specifically, upon identifying these individuals in clinical practice, an immediate and personalized plan combining intensive lifestyle intervention and mechanism-targeted pharmacotherapy should be initiated. Lifestyle modification must be weight-centric (where applicable), involving strict caloric restriction and a structured exercise program integrating both aerobic and resistance training, to directly reduce systemic lipid flux and ectopic fat deposition. Pharmacotherapy should prioritize agents that directly target key pathways in glucolipotoxicity. For instance, pioglitazone, as a potent PPAR-γ agonist, fundamentally enhances insulin sensitivity and promotes safe fatty acid storage in subcutaneous adipose tissue, thereby alleviating lipotoxicity in the liver and muscle (43). Glucagon-like peptide-1 receptor agonists (GLP-1 RAs) address the metabolic burden through multiple mechanisms including weight loss, improved glycemia, and direct reduction of hepatic and visceral fat (44). Sodium-glucose cotransporter 2 inhibitors (SGLT2i) reduce visceral and hepatic fat by promoting urinary glucose excretion, inducing a state of negative energy balance, and shifting substrate utilization, thereby improving metabolic flexibility (45). Metformin can serve as an adjuvant within this regimen. Our data suggest that initiating such a targeted, intensive combination therapy-explicitly aimed at reversing glucolipotoxicity-before these high-risk patients reach the predicted 4.0-year tipping point of accelerated decline, holds the potential to delay or even prevent the precipitous drop in insulin sensitivity. This would thereby preserve β-cell function, maintain metabolic health, and ultimately improve long-term cardiovascular and renal outcomes. Therefore, we propose that the TyG index be adopted as a routine clinical tool to identify high-risk individuals requiring this early, mechanism-driven therapeutic intensification, facilitating a paradigm shift in management from passive glucose control to proactive alteration of the disease trajectory.

### 4.4 Limitations

This study has several limitations that warrant careful consideration. First, the cross-sectional design precludes the establishment of causality. Importantly, the observed association with diabetes duration may be confounded by cohort effects, as patients diagnosed longer ago may have received different standards of care. Therefore, the proposed high-risk metabolic trajectory requires prospective validation in longitudinal studies to confirm its temporal sequence and clinical relevance.

Second, although HOMA-IR is widely used and practical for epidemiological research, it remains a surrogate index and cannot capture tissue-specific insulin sensitivity with the fidelity of hyper insulinemic-euglycemic clamp assessments. The limitations inherent in this index reinforce the need for validation of our findings using more direct or dynamic assessments of insulin sensitivity (e.g., clamp studies) in future research.

Third, the single-center nature of our study may affect generalizability. We employed robust internal measures-including standardized protocols, rigorous phenotyping, and multivariate adjustment for key confounders-to ensure internal validity. Nonetheless, patient referral patterns, regional practice styles, and local demographic characteristics may influence subgroup distributions and effect estimates. The cohort was composed of Chinese patients, and the pathophysiological patterns observed may be relevant to broader Asian populations with similar genetic and lifestyle backgrounds. However, external validation in diverse ethnic and clinical settings is essential to confirm the generalizability of our findings.

## 5 Conclusion

In conclusion, our study moves beyond establishing a simple linear relationship between diabetes duration and IR. We demonstrate that this relationship is critically modified by the patient’s baseline IR status and, most powerfully, by their TyG index. The definition of a high-risk trajectory based on a TyG index ≥ 9.187 and a diabetes duration of > 4.0 years provides a practical tool for clinicians to identify patients at imminent risk for a rapid deterioration in insulin sensitivity. This advocates for a paradigm of precision medicine in T2DM, where simple, readily available biomarkers like the TyG index can guide more intensive monitoring and tailored therapeutic strategies to improve long-term metabolic outcomes.

## Data Availability

The raw data supporting the conclusions of this article will be made available by the authors, without undue reservation.
