# Correction: The triglyceride-glucose index modulates the association between diabetes duration and insulin resistance in type 2 diabetes: a large cross-sectional study

**DOI:** 10.3389/fendo.2026.2013077

**Published:** 2026-10-13

**Authors:** Ya Dong, Xiaohong Huang, Mingyu Hao, Haiyan Li, Dewen Yan

**Affiliations:** 1 Department of Endocrinology, Shenzhen Second People’s Hospital, the First Affiliated Hospital of Shenzhen University, Health Science Center of Shenzhen University, Shenzhen Clinical Research Center for Metabolic Diseases, Shenzhen Center for Diabetes Control and Prevention, Shenzhen, China; 2 Department of Endocrinology, Shenzhen Bao’an Shiyan People’s Hospital, Bao’an Clinical Institute of Shantou University Medical College, Shenzhen, China

**Keywords:** diabetes duration, homeostatic model assessment of insulin resistance (HOMA-IR), insulin resistance (IR), triglyceride-glucose index (TyG index), type 2 diabetes mellitus (T2DM)

An incorrect **Funding** statement was provided. The correct funder is Shenzhen Science and Technology Program.

The reference for (2) was erroneously written as “GBD 2021 Diabetes Collaborators. Global, regional, and national burden of diabetes from 1990 to 2021, with projections of prevalence to 2050: a systematic analysis for the Global Burden of Disease Study 2021. Lancet. (2023) 402:203–34. doi: 10.1016/S0140-6736(23)00961-2”. It should be “GBD 2021 Diabetes Collaborators. Global, regional, and national burden of diabetes from 1990 to 2021, with projections of prevalence to 2050: a systematic analysis for the Global Burden of Disease Study 2021. Lancet. (2023) 402:203–234.doi:10.1016/S0140-6736(23)01301-6.”

The reference for (5) was erroneously written as “Ouyang Q, Zhang C, Zhang H, Xiong T, Chen Y, Zhang P. Evaluation of four insulin resistance surrogate indicators for predicting short-term mortality in critically ill patients with ischemic stroke: a nationwide retrospective cohort study. Cardiovasc Diabetol. (2025) 24:409. doi: 10.1186/s12933-025-02178-x”. It should be “Ouyang Q, Zhang C, Zhang H, Xiong T, Chen Y, Zhang P. Evaluation of four insulin resistance surrogate indicators for predicting short-term mortality in critically ill patients with ischemic stroke: a nationwide retrospective cohort study. Cardiovasc Diabetol. (2025) 24:409. doi:10.1186/s12933-025-02956-6.”

The reference for (7) was erroneously written as “Song Y, Manson JE, Tinker L, Howard BV, Kuller LH, Nathan L, et al. Insulin sensitivity and insulin secretion determined by homeostasis model assessment and risk of diabetes in a multiethnic cohort of women: the Women’s Health Initiative Observational Study. Diabetes Care. (2007) 30:1747–52. doi: 10.2337/dc07-0117.” It should be “Song Y, Manson JE, Tinker L, Howard BV, Kuller LH, Nathan L, et al. Insulin sensitivity and insulin secretion determined by homeostasis model assessment and risk of diabetes in a multiethnic cohort of women: the Women’s Health Initiative Observational Study. Diabetes Care. (2007) 30:1747-1752. doi:10.2337/dc07-0358.”

The reference for (8) was erroneously written as “Weyer C, Bogardus C, Mott DM, Pratley RE. The natural history of insulin secretory dysfunction and insulin resistance in the pathogenesis of type 2 diabetes mellitus. J Clin Invest. (1999) 104:787-794. doi: 10.1172/JCI6894.” It should be “Weyer C, Bogardus C, Mott DM, Pratley RE. The natural history of insulin secretory dysfunction and insulin resistance in the pathogenesis of type 2 diabetes mellitus. J Clin Invest. (1999) 104:787-794. doi:10.1172/JCI7231.”

The reference for (9) was erroneously written as “Tabák AG, Jokela M, Akbaraly TN, Brunner EJ, Kivimäki M, Witte DR. Trajectories of glycaemia, insulin sensitivity, and insulin secretion before diagnosis of type 2 diabetes: an analysis from the Whitehall II study. Lancet. (2009) 373:2215-21. doi: 10.1016/S0140-6736(09)60784-2.” It should be “Tabák AG, Jokela M, Akbaraly TN, Brunner EJ, Kivimäki M, Witte DR. Trajectories of glycaemia, insulin sensitivity, and insulin secretion before diagnosis of type 2 diabetes: an analysis from the Whitehall II study. Lancet. (2009) 373:2215-21. doi: 10.1016/S0140-6736(09)60619-X.”

The reference for (10) was erroneously written as “DeFronzo RA. Pathogenesis of type 2 diabetes mellitus. Med Clin North Am. (2004) 88:787–835. doi: 10.1016/j.mcna.2004.02.006.” It should be “DeFronzo RA. Pathogenesis of type 2 diabetes mellitus. Med Clin North Am. (2004) 88:787–835,ix.doi:10.1016/j.mcna.2004.04.013.”

The reference for (11) was erroneously written as Holman RR. Assessing the potential for alpha-glucosidase inhibitors in prediabetic states. Diabetes Res Clin Pract. (1998) 40 Suppl:S21-25. doi: 10.1016/S0168-8227(98)00108-6.” It should be “Holman RR. Assessing the potential for alpha-glucosidase inhibitors in prediabetic states. Diabetes Res Clin Pract. (1998) 40 Suppl:S21-25. doi: 10.1016/s0168-8227(98)00038-2.”

The reference for (12) was erroneously written as “Poitout V, Robertson RP. Glucolipotoxicity: fuel excess and beta-cell dysfunction. Endocr Rev. (2008) 29:351–66. doi: 10.1210/er.2007-0022.” It should be “Poitout V, Robertson RP. Glucolipotoxicity: fuel excess and beta-cell dysfunction. Endocr Rev. (2008) 29:351-66. doi: 10.1210/er.2007-0023.”

The reference for (14) was erroneously written as “Wu WC, Wei JN, Chen SC, Fan KC, Lin CH, Yang CY, et al. Progression of insulin resistance: A link between risk factors and the incidence of diabetes. Diabetes Res Clin Pract. (2020) 161:108050. doi: 10.1016/j.diabres.2019.108050.” It should be “Wu WC, Wei JN, Chen SC, Fan KC, Lin CH, Yang CY, et al. Progression of insulin resistance: A link between risk factors and the incidence of diabetes. Diabetes Res Clin Pract. (2020) 161:108050. doi: 10.1016/j.diabres.2020.108050”.

The reference for (16) was erroneously written as “Li ZF, Yin Z, Li X, Lu MY, Zhang WJ, Luo F, et al. Effect of changes in the triglyceride-glucose index on atherogenic lipid profiles in coronary artery disease patients receiving lipid-lowering therapy: a prospective cohort study. Cardiovasc Diabetol. (2025) 24:372. doi: 10.1186/s12933-025-02134-0“. It should be “Li ZF, Yin Z, Li X, Lu MY, Zhang WJ, Luo F, et al. Effect of changes in the triglyceride-glucose index on atherogenic lipid profiles in coronary artery disease patients receiving lipid-lowering therapy: a prospective cohort study. Cardiovasc Diabetol. (2025) 24:372. doi: 10.1186/s12933-025-02910-6.”

The reference for (17) was erroneously written as “Wang M, Mei L, Jin A, Cai X, Jing J, Wang S, et al. Association between triglyceride glucose index and atherosclerotic plaques and Burden: findings from a community-based study. Cardiovasc Diabetol. (2022) 21:204. doi: 10.1186/s12933-022-01557-1.” It should be “Wang M, Mei L, Jin A, Cai X, Jing J, Wang S, et al. Association between triglyceride glucose index and atherosclerotic plaques and Burden: findings from a community-based study. Cardiovasc Diabetol. (2022) 21:204. doi: 10.1186/s12933-022-01638-x.”

The reference for (20) was erroneously written as Cheng J, Cheng Q, Wu Y, Yin J, He F. Association between triglyceride-glucose-body mass index and adverse prognosis in elderly patients with severe heart failure and type 2 diabetes: a retrospective study based on the MIMIC-IV database. Cardiovasc Diabetol. (2025) 24:299. doi: 10.1186/s12933-025-02093-5. It should be Cheng J, Cheng Q, Wu Y, Yin J, He F. Association between triglyceride-glucose-body mass index and adverse prognosis in elderly patients with severe heart failure and type 2 diabetes: a retrospective study based on the MIMIC-IV database. Cardiovasc Diabetol. (2025) 24:299. doi: 10.1186/s12933-025-02870-x.”

The reference for (23) was erroneously written as “World Health Organization. Definition, Diagnosis, and Classification of Diabetes Mellitus and its Complications. Part 1. Diagnosis and Classification of Diabetes Mellitus. Geneva: World Health Organization; 1999.” It should be “Alberti KG, Zimmet PZ. Definition, diagnosis and classification of diabetes mellitus and its complications. Part 1: diagnosis and classification of diabetes mellitus provisional report of a WHO consultation. Diabet Med. (1998) 15:539-53. doi: 10.1002/(SICI)1096-9136(199807)15:7<539::AID-DIA668>3.0.CO;2-S.”

The reference for (24) was erroneously written as “Chen L, Jia WP, Lu JQ, Bao YQ, Wu YM, Jiang SY, et al. Shanghai adult metabolic syndrome prevalence survey. Chin J Cardiol. (2003) 31:909–12. doi:10.3760/j:issn:0253-3758.2003.12.007.” It should be “Chen L, Jia WP, Lu JQ, Bao YQ, Wu YM, Jiang SY, et al. Prevalence of metabolic syndrome among Shanghai adults in China. Chin J Cardiol. (2003) 31:909–912. doi:10.3760/j:issn:0253-3758.2003.12.007.”

The reference for (25) was erroneously written as “Defronzo RA. From the triumvirate to the ominous octet: a new paradigm for the treatment of type 2 diabetes mellitus. Diabetes. (2009) 58:773-95. doi: 10.2337/db08-1424.” It should be “Defronzo RA. Banting Lecture. From the triumvirate to the ominous octet: a new paradigm for the treatment of type 2 diabetes mellitus. Diabetes. (2009) 58:773–95.doi: 10.2337/db09-9028.”

The reference for (29) was erroneously written as “Ding L, Zhang H, Liu Y, Jiang Z, Yu F, Qi Y, et al. Associations between triglyceride-glucose-related indices and all-cause and cardiovascular mortality among individuals with atrial fibrillation and metabolic syndrome: a cohort study of the UK biobank. Cardiovasc Diabetol. (2025) 24:414. doi: 10.1186/s12933-025-02182-4.” It should be “Ding L, Zhang H, Liu Y, Jiang Z, Yu F, Qi Y, et al. Associations between triglyceride-glucose-related indices and all-cause and cardiovascular mortality among individuals with atrial fibrillation and metabolic syndrome: a cohort study of the UK biobank. Cardiovasc Diabetol. (2025) 24:414. doi: 10.1186/s12933-025-02963-7.”

The reference for (30) was erroneously written as “Simental-Mendía LE, Rodríguez-Morán M, Guerrero-Romero F. The product of fasting glucose and triglycerides as surrogate for identifying insulin resistance in apparently healthy subjects. Metab Syndr Relat Disord. (2008) 6:299-304. doi: 10.1089/met.2008.0039.”

It should be “Simental-Mendía LE, Rodríguez-Morán M, Guerrero-Romero F. The product of fasting glucose and triglycerides as surrogate for identifying insulin resistance in apparently healthy subjects. Metab Syndr Relat Disord. (2008) 6:299-304. doi: 10.1089/met.2008.0034.”

The reference for (31) was erroneously written as “Lai Y, Lin C, Liu X, Liu Y, Cai H, Zhao N, et al. Association of triglyceride-glucose index trajectories with the risk of worsening heart failure in elderly patients with chronic heart failure and type 2 diabetes: a competing risk analysis. Cardiovasc Diabetol. (2025) 24:131. doi: 10.1186/s12933-025-02029-6.” It should be “Lai Y, Lin C, Liu X, Liu Y, Cai H, Zhao N, et al. Association of triglyceride-glucose index trajectories with the risk of worsening heart failure in elderly patients with chronic heart failure and type 2 diabetes: a competing risk analysis. Cardiovasc Diabetol. (2025) 24:131. doi: 10.1186/s12933-025-02687-8.”

The reference for (36) was erroneously written as “Zhang Q, Xiao S, Jiao X, Shen Y. The triglyceride-glucose index is a predictor for cardiovascular and all-cause mortality in CVD patients with diabetes or pre-diabetes: evidence from NHANES 2001-2018. Cardiovasc Diabetol. (2023) 22:279. doi: 10.1186/s12933-023-01762-2.” It should be “Zhang Q, Xiao S, Jiao X, Shen Y. The triglyceride-glucose index is a predictor for cardiovascular and all-cause mortality in CVD patients with diabetes or pre-diabetes: evidence from NHANES 2001-2018. Cardiovasc Diabetol. (2023) 22:279. doi: 10.1186/s12933-023-02030-z.”

The reference for (37) was erroneously written as “Zhao J, Li N, Li S, Dou J. The predictive significance of the triglyceride-glucose index in forecasting adverse cardiovascular events among type 2 diabetes mellitus patients with co-existing hyperuricemia: a retrospective cohort study. Cardiovasc Diabetol. (2025) 24:218. doi: 10.1186/s12933-025-02062-5.” It should be “Zhao J, Li N, Li S, Dou J. The predictive significance of the triglyceride-glucose index in forecasting adverse cardiovascular events among type 2 diabetes mellitus patients with co-existing hyperuricemia: a retrospective cohort study. Cardiovasc Diabetol. (2025) 24:218. doi: 10.1186/s12933-025-02783-9.”

The reference for (38) was erroneously written as “Wu L, Bao X, Xu J, Ma L, Kang L, Zhang R. The triglyceride-glucose index positively associates with the prevalence and severity of coronary heart disease in patients among hypertension. Sci Rep. (2025) 15:19571. doi: 10.1038/s41598-025-07524-3.” It should be “Wu L, Bao X, Xu J, Ma L, Kang L, Zhang R. The triglyceride-glucose index positively associates with the prevalence and severity of coronary heart disease in patients among hypertension. Sci Rep. (2025) 15:19571. doi: 10.1038/s41598-025-03948-y.”

The reference for (39) was erroneously written as “Xu F, Feng Y, Zhong X. Higher triglyceride glucose index is associated with increased risk of stroke among middle-aged and elderly Chinese: a national longitudinal study. Sci Rep. (2024) 14:19054. doi: 10.1038/s41598-024-61104-7.” It should be “Xu F, Feng Y, Zhong X. Higher triglyceride-glucose index is associated with increased risk of stroke among middle-aged and elderly Chinese: a national longitudinal study. Sci Rep. (2024) 14:19054. doi: 10.1038/s41598-024-70008-2.”

The reference for (40) was erroneously written as “Davies MJ, Aroda VR, Collins BS, Gabbay RA, Green J, Maruthur NM, et al. Management of hyperglycemia in type 2 diabetes, 2022. A consensus report by the american diabetes association (ADA) and the european association for the study of diabetes (EASD). Diabetes Care. (2022) 45:2753–86. doi: 10.2337/dc22-0062.” It should be “Davies MJ, Aroda VR, Collins BS, Gabbay RA, Green J, Maruthur NM, et al. Management of hyperglycemia in type 2 diabetes, 2022. A consensus report by the american diabetes association (ADA) and the european association for the study of diabetes (EASD). Diabetes Care. (2022) 45:2753–86. doi: 10.2337/dci22-0034.”

The reference for (41) was erroneously written as “ElSayed NA, Aleppo G, Aroda VR, Bannuru RR, Brown FM, Bruemmer D, et al. Pharmacologic approaches to glycemic treatment: standards of care in diabetes-2023. Diabetes Care. (2023) 46:S140–57. doi: 10.2337/dc23-S009.” It should be “ElSayed NA, Aleppo G, Aroda VR, Bannuru RR, Brown FM, Bruemmer D, et al. 9. Pharmacologic approaches to glycemic treatment: standards of care in diabetes-2023. Diabetes Care. (2023) 46:S140–S157. doi:10.2337/dc23-S009.

”The reference for (43) was erroneously written as “Rotman Y, Sanyal AJ. Current and upcoming pharmacotherapy for non-alcoholic fatty liver disease. Gut. (2017) 66:180–90. doi: 10.1136/gutjnl-2016-311828.” It should be “Rotman Y, Sanyal AJ. Current and upcoming pharmacotherapy for non-alcoholic fatty liver disease. Gut. (2017) 66:180–90. doi: 10.1136/gutjnl-2016-312431.”

The reference for (44) was erroneously written as “Drucker DJ. GLP-1-based therapies for diabetes, obesity and beyond. Nat Rev Drug Discov. (2025) 24:631–50. doi:10.1038/s41573-025-00786-7.” It should be “Drucker DJ. GLP-1-based therapies for diabetes, obesity and beyond. Nat Rev Drug Discov. (2025) 24:631–50. doi: 10.1038/s41573-025-01183-8.”

The reference for (45) was erroneously written as “Latva-Rasku A, Rebelos E, Tuisku J, Aarnio R, Bhowmik A, Keskinen H, et al. SGLT2 inhibitor dapagliflozin increases skeletal muscle and brain fatty acid uptake in individuals with type 2 diabetes: A randomized double-blind placebo-controlled positron emission tomography study. Diabetes Care. (2024) 47:1630–7. doi: 10.2337/dc24-0312.” It should be “Latva-Rasku A, Rebelos E, Tuisku J, Aarnio R, Bhowmik A, Keskinen H, et al. SGLT2 inhibitor dapagliflozin increases skeletal muscle and brain fatty acid uptake in individuals with type 2 diabetes: A randomized double-blind placebo-controlled positron emission tomography study. Diabetes Care. (2024) 47:1630–7. doi: 10.2337/dc24-0470.”

Three incorrect in-text citations were identified in the article.

1. In the **Discussion** section, subsection 4.1 *The Positioning within a precision-phenotyping framework*, first paragraph, the citation was erroneously written as (22–25). The correct citation is (25).

The sentence was erroneously written as:

“The independent positive association between diabetes duration and HOMA-IR in our overall cohort aligns with the natural history of T2DM, where progressive IR often occurs over time (22-25).” It should read: “The independent positive association between diabetes duration and HOMA-IR in our overall cohort aligns with the natural history of T2DM, where progressive IR often occurs over time (25).”

2. In the **Discussion** section, subsection The TyG Index: A Pivotal Stratifier and Potential Driver of High-Risk Trajectories, first paragraph, the citation was erroneously written as (26,31). The correct citation is (31).

The sentence was erroneously written as: “A high TyG index signifies a state of pronounced lipid dysregulation (elevated triglycerides, often with low HDL-C) and glucose intolerance (26,31).” It should read: “A high TyG index signifies a state of pronounced lipid dysregulation (elevated triglycerides, often with low HDL-C) and glucose intolerance (31).”

3. In the **Discussion** section, subsection *The High-Risk Trajectory: Clinical Implications for Precision Medicine*, second paragraph, the citation was erroneously written as (39–41). The correct citation is (40,41).

The sentence was erroneously written as:

“Current clinical management of type 2 diabetes is primarily guided by individualized glycemic targets, body weight, and established cardiovascular, kidney, and other metabolic comorbidities, while simple and practical tools for prospectively identifying patients at particularly high risk of accelerated progression of insulin resistance are not routinely incorporated into current treatment algorithms (39-41).” It should read: “Current clinical management of type 2 diabetes is primarily guided by individualized glycemic targets, body weight, and established cardiovascular, kidney, and other metabolic comorbidities, while simple and practical tools for prospectively identifying patients at particularly high risk of accelerated progression of insulin resistance are not routinely incorporated into current treatment algorithms (40,41).”

The statement “Current clinical guidelines primarily recommend initiating intensive therapy only after metabolic control has significantly deteriorated (39–41), an inherently reactive strategy that lacks reliable tools to proactively identify high-risk patients. This approach is concerning,” inaccurately characterized current clinical management of type 2 diabetes.

A correction has been made to the section 4 **Discussion**, 4.3 *The High-Risk Trajectory: Clinical Implications for Precision Medicine*, second paragraph:

“Current clinical management of type 2 diabetes is primarily guided by individualized glycemic targets, body weight, and established cardiovascular, kidney, and other metabolic comorbidities, while simple and practical tools for prospectively identifying patients at particularly high risk of accelerated progression of insulin resistance are not routinely incorporated into current treatment algorithms (40,41). This represents an important clinical gap,”

The original version of this article has been updated.

