# Supplementary material for: The triglyceride-glucose index modulates the association between diabetes duration and insulin resistance in type 2 diabetes: a large cross-sectional study
Source: Front Endocrinol (Lausanne). 2026 Feb 18;17:1762526. doi: 10.3389/fendo.2026.1762526 (PMC12956789; doi:10.3389/fendo.2026.1762526)
Supplement: Supplementary file 1 [file Table1.docx]

**Supplement data**

**Table S1. Associations of diabetes duration and relevant covariates with HOMA-IR in patients with T2DM across different models**

| Variables | Model1 | | |  | Model2 | | |  | Model3 | | |
| --- | --- | --- | --- | --- | --- | --- | --- | --- | --- | --- | --- |
|  | β (95% CI) | *P* value | Adj_R^2^ |  | β (95% CI) | *P* value | Adj_R^2^ |  | β (95% CI) | *P* value | Adj_R^2^ |
| Diabetes duration (years) | 0.06 (0.05, 0.08) | < 0.001 | 0.02 |  | 0.07 (0.06, 0.09) | < 0.001 | 0.07 |  | 0.04 (0.03, 0.05) | < 0.001 | 0.33 |
| Age (years) | - | - |  |  | -0.01 (-0.02, 0.00) | 0.061 |  |  | 0.01 (-0.00, 0.02) | 0.055 |  |
| Female | - | - |  |  | 0.83 (0.62, 1.04) | < 0.001 |  |  | 0.72 (0.52, 0.92) | < 0.001 |  |
| BMI (kg/m^2^) | - | - |  |  | 0.04 (-0.01, 0.08) | 0.127 |  |  | 0.05 (0.00, 0.09) | 0.033 |  |
| WC (cm) | - | - |  |  | 0.05 (0.03, 0.07) | < 0.001 |  |  | 0.02 (0.01, 0.03) | 0.008 |  |
| HbA1c (%) | - | - |  |  | - | - |  |  | 0.33 (0.29, 0.37) | < 0.001 |  |
| TyG index | - | - |  |  | - | - |  |  | 1.53 (1.34, 1.72) | < 0.001 |  |
| Lymphocyte count (10^9^/L) | - | - |  |  | - | - |  |  | 0.02 (-0.15, 0.20) | 0.794 |  |
| DR | - | - |  |  | - | - |  |  | 0.23 (0.05, 0.41) | 0.014 |  |
| MAFLD | - | - |  |  | - | - |  |  | 0.10 (-0.08, 0.28) | 0.294 |  |
| Insulin use | - | - |  |  | - | - |  |  | 1.28 (1.10, 1.46) | < 0.001 |  |
| HDL-C (mmol/L) | - | - |  |  | - | - |  |  | 0.06 (-0.26, 0.39) | 0.708 |  |
| ALT (U/L) | - | - |  |  | - | - |  |  | 0.01 (0.00, 0.01) | 0.005 |  |
| GGT (U/L) | - | - |  |  | - | - |  |  | 0.00 (-0.00, 0.00) | 0.933 |  |
| CRP (mg/L) | - | - |  |  | - | - |  |  | 0.01 (0.00, 0.02) | 0.008 |  |
| Neutrophil count (10^9^/L) | - | - |  |  | - | - |  |  | -0.00 (-0.12, 0.11) | 0.939 |  |
| Hypertension | - | - |  |  | - | - |  |  | 0.01 (-0.17, 0.18) | 0.947 |  |
| Coronary heart disease | - | - |  |  | - | - |  |  | 0.39 (0.13, 0.64) | 0.003 |  |
| DN | - | - |  |  | - | - |  |  | 0.04 (-0.17, 0.25) | 0.686 |  |
| TG (mmol/L) | - | - |  |  | - | - |  |  | -0.35 (-0.42, -0.28) | < 0.001 |  |
| WBC (10^9^/L) | - | - |  |  | - | - |  |  | 0.05 (-0.06, 0.15) | 0.364 |  |
| DPN | - | - |  |  | - | - |  |  | -0.14 (-0.32, 0.04) | 0.140 |  |
| Scr (umol/L) | - | - |  |  | - | - |  |  | 0.01 (0.00, 0.01) | < 0.001 |  |

**Table S2. Association between diabetes duration and HOMA-IR, stratified by baseline insulin resistance status**

| Variables | Model1 | | |  | | Model2 | | |  | Model3 | | |
| --- | --- | --- | --- | --- | --- | --- | --- | --- | --- | --- | --- | --- |
|  | β (95% CI) | *P* value | Adj_R^2^ |  | | β (95% CI) | *P* value | Adj_R^2^ |  | β (95% CI) | *P* value | Adj_R^2^ |
| non-IR group |  |  |  |  |  | |  |  |  |  |  |  |
| Diabetes duration (years) | -0.00 (-0.01, 0.00) | 0.642 | 0.00 |  | | 0.00 (-0.00, 0.01) | 0.072 | 0.09 |  | 0.00 (-0.00, 0.01) | 0.211 | 0.17 |
| Age (years) | - | - |  |  | | -0.00 (-0.01, -0.00) | 0.003 |  |  | 0.00 (-0.00, 0.00) | 0.779 |  |
| Female | - | - |  |  | | 0.07 (0.01, 0.13) | 0.021 |  |  | 0.06 (-0.01, 0.12) | 0.080 |  |
| BMI (kg/m^2^) | - | - |  |  | | 0.02 (0.01, 0.03) | 0.003 |  |  | 0.02 (0.01, 0.03) | 0.006 |  |
| WC (cm) | - | - |  |  | | 0.01 (0.01, 0.02) | < 0.001 |  |  | 0.01 (0.00, 0.01) | 0.002 |  |
| HbA1c (%) | - | - |  |  | | - | - |  |  | 0.04 (0.02, 0.05) | < 0.001 |  |
| TyG index | - | - |  |  | | - | - |  |  | 0.14 (0.07, 0.20) | < 0.001 |  |
| Lymphocyte count (10^9^/L) | - | - |  |  | | - | - |  |  | 0.05 (-0.01, 0.10) | 0.076 |  |
| DR | - | - |  |  | | - | - |  |  | -0.01 (-0.06, 0.05) | 0.732 |  |
| MAFLD | - | - |  |  | | - | - |  |  | 0.07 (0.02, 0.13) | 0.013 |  |
| Insulin use | - | - |  |  | | - | - |  |  | 0.16 (0.11, 0.21) | < 0.001 |  |
| HDL-C (mmol/L) | - | - |  |  | | - | - |  |  | -0.04 (-0.13, 0.06) | 0.468 |  |
| ALT (U/L) | - | - |  |  | | - | - |  |  | 0.00 (0.00, 0.01) | < 0.001 |  |
| GGT (U/L) | - | - |  |  | | - | - |  |  | 0.00 (-0.00, 0.00) | 0.117 |  |
| CRP (mg/L) | - | - |  |  | | - | - |  |  | 0.00 (-0.00, 0.00) | 0.902 |  |
| Neutrophil count (10^9^/L) | - | - |  |  | | - | - |  |  | 0.01 (-0.02, 0.05) | 0.426 |  |
| Hypertension | - | - |  |  | | - | - |  |  | 0.03 (-0.03, 0.08) | 0.316 |  |
| Coronary heart disease | - | - |  |  | | - | - |  |  | 0.04 (-0.04, 0.12) | 0.341 |  |
| DN | - | - |  |  | | - | - |  |  | 0.02 (-0.04, 0.09) | 0.483 |  |
| TG (mmol/L) | - | - |  |  | | - | - |  |  | -0.03 (-0.05, -0.00) | 0.024 |  |
| WBC (10^9^/L) | - | - |  |  | | - | - |  |  | -0.01 (-0.04, 0.02) | 0.686 |  |
| DPN | - | - |  |  | | - | - |  |  | -0.04 (-0.09, 0.01) | 0.134 |  |
| Scr (umol/L) | - | - |  |  | | - | - |  |  | -0.00 (-0.00, 0.00) | 0.428 |  |
| IR group |  |  |  |  | |  |  |  |  |  |  |  |
| Diabetes duration (years) | 0.05 (0.04, 0.07) | < 0.001 | 0.02 |  | | 0.05 (0.03, 0.07) | < 0.001 | 0.04 |  | 0.04 (0.02, 0.06) | < 0.001 | 0.18 |
| Age (years) | - | - |  |  | | -0.00 (-0.01, 0.01) | 0.904 |  |  | 0.01 (-0.00, 0.02) | 0.151 |  |
| Female | - | - |  |  | | 0.75 (0.46, 1.03) | < 0.001 |  |  | 0.68 (0.39, 0.96) | < 0.001 |  |
| BMI (kg/m^2^) | - | - |  |  | | -0.04 (-0.11, 0.02) | 0.166 |  |  | -0.20 (-0.08, 0.04) | 0.520 |  |
| WC (cm) | - | - |  |  | | 0.04 (0.02, 0.06) | < 0.001 |  |  | 0.02 (0.00, 0.05) | 0.022 |  |
| HbA1c (%) | - | - |  |  | | - | - |  |  | 0.19 (0.13, 0.25) | < 0.001 |  |
| TyG index | - | - |  |  | | - | - |  |  | 1.60 (1.33, 1.86) | < 0.001 |  |
| Lymphocyte count (10^9^/L) | - | - |  |  | | - | - |  |  | -0.14 (-0.40, 0.11) | 0.266 |  |
| DR | - | - |  |  | | - | - |  |  | 0.22 (-0.05, 0.49) | 0.103 |  |
| MAFLD | - | - |  |  | | - | - |  |  | 0.05 (-0.21, 0.32) | 0.696 |  |
| Insulin use | - | - |  |  | | - | - |  |  | 1.05 (0.75, 1.34) | < 0.001 |  |
| HDL-C (mmol/L) | - | - |  |  | | - | - |  |  | 0.04 (-0.43, 0.52) | 0.864 |  |
| ALT (U/L) | - | - |  |  | | - | - |  |  | 0.00 (-0.01, 0.01) | 0.690 |  |
| GGT (U/L) | - | - |  |  | | - | - |  |  | -0.00 (-0.00, 0.00) | 0.984 |  |
| CRP (mg/L) | - | - |  |  | | - | - |  |  | 0.01 (-0.00, 0.02) | 0.125 |  |
| Neutrophil count (10^9^/L) | - | - |  |  | | - | - |  |  | 0.06 (-0.12, 0.24) | 0.497 |  |
| Hypertension | - | - |  |  | | - | - |  |  | -0.06 (-0.32, 0.20) | 0.644 |  |
| Coronary heart disease | - | - |  |  | | - | - |  |  | 0.29 (-0.07, 0.65) | 0.115 |  |
| DN | - | - |  |  | | - | - |  |  | -0.02 (-0.32, 0.27) | 0.868 |  |
| TG (mmol/L) | - | - |  |  | | - | - |  |  | -0.36 (-0.45, -0.27) | < 0.001 |  |
| WBC (10^9^/L) | - | - |  |  | | - | - |  |  | 0.03(-0.13, 0.18) | 0.753 |  |
| DPN | - | - |  |  | | - | - |  |  | -0.21 (-0.48, 0.07) | 0.144 |  |
| Scr (umol/L) | - | - |  |  | | - | - |  |  | 0.00 (-0.00, 0.01) | 0.095 |  |
